# 

**Published:** 1889-11

**Authors:** 


					﻿$2.00 A YEAR IN ADVANCE.
Whole Number,
CCCXL.
New Series.
1889,
VOL. XXIX..
No. 4.
Buffalo
Medical //Surgical
Journal.
Established 1845 by AUSTIN FLINT, NT. D.
Sr&itors:
THOS. LOTHROP, M. D.
W. W. POTTER, M. D.
Associate Suitors:
FRANK HAMILTON POTTER, M. D.
WILLIAM C. KRAUSS, M. D.
JAMES WRIGHT PUTNAM, M. D.
JOHN PARMENTER, M. D.
BUFFALO:
1889.
Entered at the Post-Office at Buffalo, N. Y., as Second-class Mail Matter.
Times Printing House, Buffalo, N. X
				

